# Three-Dimensional Reconstructed Bone Marrow Matrix Culture Improves the Viability of Primary Myeloma Cells In-Vitro via a STAT3-Dependent Mechanism

**DOI:** 10.3390/cimb43010026

**Published:** 2021-06-08

**Authors:** Yung-Hsing Huang, Meaad Almowaled, Jing Li, Christopher Venner, Irwindeep Sandhu, Anthea Peters, Afsaneh Lavasanifar, Raymond Lai

**Affiliations:** 1 Department of Laboratory Medicine and Pathology, University of Alberta, Edmonton, AB T6G 2E1, Canada; yunghsin@ualbeta.ca (Y.-H.H.); 2489203a@student.gla.ac.uk (M.A.); lijing2020@hrbmu.edu.cn (J.L.); 2 Electron Microscopy Center, Basic Medical Science College, Harbin Medical University, Harbin 150080, China; 3 Department of Oncology, University of Alberta, Edmonton, AB T6G 2R7, Canada; cvenner@ualberta.ca (C.V.); irwindee@ualberta.ca (I.S.); anthea1@ualberta.ca (A.P.); 4 Department of Medicine, University of Alberta, Edmonton, AB T6G 2G3, Canada; 5 Faculty of Pharmacy and Pharmaceutical Sciences, University of Alberta, Edmonton, AB T6G2H7, Canada; afsaneh@ualberta.ca

**Keywords:** 3D culture, primary myeloma cells, STAT3

## Abstract

Primary myeloma (PM) cells are short-lived in conventional culture, which limited their usefulness as a study model. Here, we evaluated if three-dimensional (3D) culture can significantly prolong the longevity of PM cells in-vitro. We employed a previously established 3D model for culture of bone marrow mononuclear cells isolated from 15 patients. We assessed the proportion of PM cells, viability and proliferation using CD38 staining, trypan blue exclusion assays and carboxy fluorescein succinimidyl ester (CFSE) staining, respectively. We observed significantly more CD38+ viable cells in 3D than in conventional culture (65% vs. 25%, *p* = 0.006) on day 3. CFSE staining showed no significant difference in cell proliferation between the two culture systems. Moreover, we found that PM cells in 3D culture are more STAT3 active by measure of pSTAT3 staining (66% vs. 10%, *p* = 0.008). Treatment of IL6, a STAT3 activator significantly increased CD38+ cell viability (41% to 68%, *p* = 0.021). In comparison, inhibition of STAT3 with Stattic significantly decreased PM cell viability in 3D culture (38% to 17% *p* = 0.010). Neither IL6 nor Stattic affected the PM cell viability in conventional culture. This study suggests that 3D culture can significantly improve the longevity of PM cells in-vitro, and STAT3 activation can further improve their viability.

## 1. Introduction

Although immortalized human cancer cell lines have advanced cancer research since the 1950s, their suitability to represent real tumors has been challenged. To further complicate the issue, it was reported that 18% to 46% of human cancer cell lines are misidentified or cross-contaminated over time [1,2]. Several comparative studies between primary cells and cell lines had reported remarkable differences in their gene expression profiles [3,4,5]. Human cancer cell lines also may harbor gene mutations which are not seen in their respective natural diseases [6]. In myeloma studies, a proteomic analysis comparing primary myeloma (PM) cells and myeloma cell lines showed significant differences in the expressions of biosynthesis proteins (such as ribosomal subunits, chaperons and translational factors) and immune response elements (such as complement receptors and MHC molecules) [7]. It also had been pointed out that the use of myeloma cell lines do not usually take into account of the tumor microenvironment, which is essential for the support of the survival and growth of myeloma cells in patients [8]. PM cells have been used for the preclinical evaluation of novel therapeutic agents such as daratumumab [9,10]. Nonetheless, the sources for PM cells are relatively limited. More important, it is well-known that PM cells, without appropriate support, remain viable in conventional culture for limited time (<7 days) [11].

The rapid loss of cell viability of PM cells kept in conventional culture was found even in the presence of stimulating cytokines (e.g., IL6) and growth factors (e.g., IGF1), soluble factors that are known to induce the in-vitro growth of myeloma cell lines [11]. In an attempt to overcome this technical challenge, researchers have experimented on the use of three-dimensional (3D) culture systems. Compared to conventional culture, 3D culture models allow tumor cells to form tumor-like spheroids in a matrix that resembles human stroma, providing a favorable growth environment [12]. In one study, reconstructed bone marrow matrix cell culture method was shown to maintain both the PM cells and bone marrow stromal cells for up to 30 days [13], but this method has not been fully adopted to our best knowledge. The molecular basis underlying the benefits of using 3D culture is largely unknown. In this regard, we have previously shown that STAT3 signaling was activated in myeloma cell lines cultured in 3D [14]. Moreover, we found that 3D-cultured myeloma cell lines underwent apoptosis after STAT3 inhibition. STAT3 is hyperactive in approximately 50% of MM patients [15,16,17], and inhibition of STAT3 activity promotes apoptosis in MM cells [18]. Taken together, our findings have led us to hypothesize that STAT3 activity is a key factor in maintaining the viability of PM cells cultured in 3D.

In this study, we first tested if the 3D culture system we used previously can significantly increase the viability of PM cells in-vitro, as compared to conventional culture. To test our hypothesis that STAT3 activity is a key factor in maintaining the viability of 3D cultured PM cells, we used flow cytometry to quantify the expression of the activated/phosphorylated form of STAT3 (pSTAT3). Additionally, we modulated the STAT3 activity to examine its effects on the viability in PM cells.

## 2. Results

### 2.1. The Viability of Primary MM Cells Is Higher in 3D Culture

Firstly, we asked whether the viability of PM cells can be improved by using the 3D culture system we employed previously [14]. We cultured bone marrow aspirate samples harvested from 15 consecutive MM patients in 3D as well as conventional culture. For each bone marrow sample, we employed flow cytometry to assess the percentage of CD38+ cells on day 3, 7 and 10. In addition, the cell viability for each sample was assessed by performing trypan blue exclusion assay. The absolute viable PM cell number was then calculated by multiplying the percentage of CD38+ cells and the total number of viable cells.

**Figure 1:**
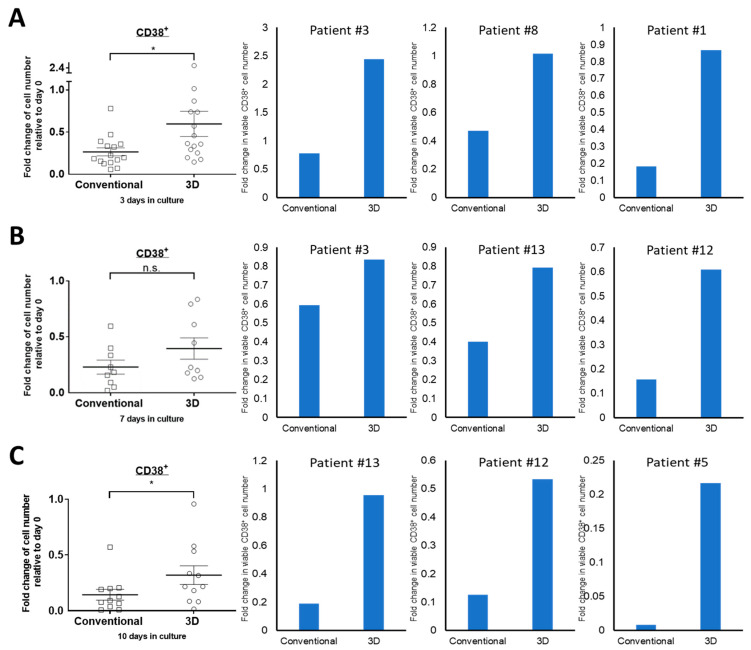
PM cells are better preserved in 3D culture compared to conventional culture. Fold change of viable CD38+ cell numbers from MM patients in conventional or 3D culture on (**A**) day 3 (*n* = 15), (**B**) day 7 (*n* = 11) and (**C**) day 10 (*n* = 11) relative to day 0. The mean values were indicated with error bars representing standard error. * *p* < 0.05, paired Student’s *t*-test.

On day 3, in 13 out of 15 MM samples, the numbers of viable CD38+ MM cells cultured in 3D culture are significantly higher than those cultured in conventional culture (Figure 1A). In the remaining 2 cases, the viable CD38 cell numbers are not statistically different between the two culture systems. Overall, the 3D group showed significantly higher viability than the conventional culture group (65% vs. 25%, *p* = 0.006). This difference remained to be statistically significant for day 7 and day 10 (36% vs. 21%, *p* = 0.028; 32% vs. 14%, *p* = 0.029, respectively, Figure 1B,C). Since the most dramatic difference between the two culture systems was seen on day 3, we used this time point for further experiments.

### 2.2. The Proliferation Rate of MM Cells Is Similar between 3D and Conventional Culture

**Figure 2:**
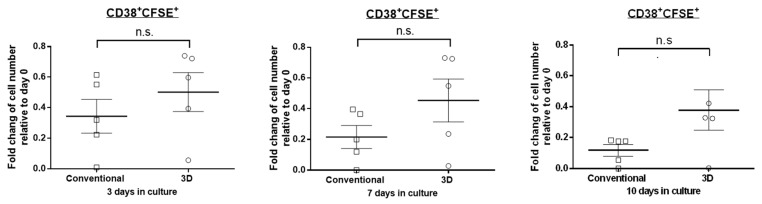
PM cells in 3D did not proliferate more rapidly than those in conventional culture. Fold change of viable CD38 + CFSE+ cell number from 5 MM patients in conventional or 3D culture on day 3, 7 and 10 relative to day 0. The mean values were indicated with error bars representing standard error. n.s. not significant, paired Student’s *t*-test.

We next asked if the better preservation of cell viability in 3D was due to a higher rate of cell proliferation. Thus, we employed the CFSE staining assay to quantify the proliferation rate in PM cells cultured in the two different systems. In principle, the CFSE staining is expected to decrease as the cells divide over time. As shown in Figure 2, we found that in five of five cases, there is no significant difference in the fold change of CD38 + CFSE+ cells between the two culture systems on day 3 (34% and 50% of day 0 for conventional and 3D culture, respectively, *p* = 0.300, paired Student’s *t*-test). A similar finding was observed on day 7 and day 10 (*p* = 0.128 and *p* = 0.076, respectively, paired Student’s *t*-test). This finding suggests that the superior PM cell viability in 3D culture is not due to a higher cell proliferation rate relative to conventional culture.

### 2.3. STAT3 Is More Active in PM Cells Cultured in 3D Culture

**Figure 3:**
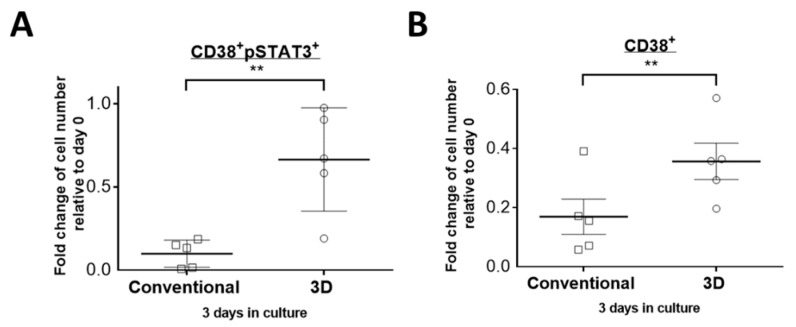
STAT3 activity is higher in PM cells cultured in 3D. Fold change of viable (**A**) CD38 + pSTAT3+ and (**B**) CD38+ cell number from 5 MM patients in conventional or 3D culture on day 3 relative to day 0. The mean value was indicated with error bars representing standard error. ** *p* < 0.001, paired Student’s *t*-test.

We previously reported that the STAT3 activity is essential for the cell viability of two myeloma cell lines (U266 and RPMI8226) in 3D but not in conventional culture [14]. In light of these findings, we hypothesized that the STAT3 activity also plays a role in maintaining the relatively high PM cell viability in 3D culture. To test this hypothesis, we assessed the STAT3 activity in PM cells by double-staining the bone marrow mononuclear cells with anti-CD38 and anti-pSTAT3 (Y705) antibodies. As shown in Figure 3A, we found significantly more viable CD38 + pSTAT3+ cells present in 3D culture on day 3 when compared to conventional culture. Specifically, in five of five samples, 66% of viable CD38 + pSTAT3+ cell number remained in 3D, compared to only 10% in conventional culture (*p* = 0.008, paired Student’s *t*-test). In the same experiment, these five samples showed a significantly higher CD38+ cell viability in 3D culture than conventional culture (36% vs. 17%, *p* = 0.003, paired Student’s *t*-test) as shown in Figure 3B.

### 2.4. IL6 Further Improved PM Cell Viability in 3D but Not Conventional Culture

**Figure 4:**
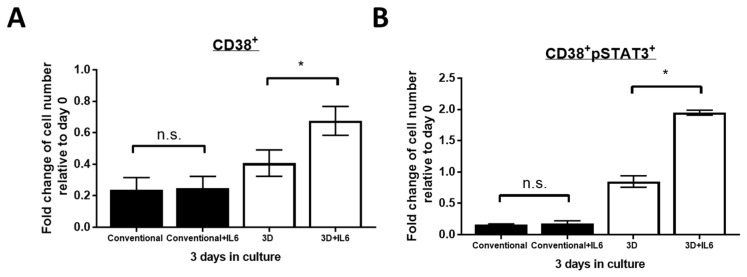
IL6 improved PM cell viability in 3D but not in conventional culture. Fold change of viable (**A**) CD38+ and (**B**) CD38 + pSTAT3+ cell number from 3 MM patients in conventional or 3D culture on day 3 relative to day 0. 30 pg/mL IL6 was added to cells every 24 h for 3 days. The mean value was indicated with error bars representing standard error. n.s. not significant, * *p* < 0.05, paired Student’s *t*-test.

We next asked if the addition of IL6, a well-known STAT3 activating cytokine in myeloma [13], will help improve CD38+ cell viability. We treated the bone marrow mononuclear cells cultured in 3D or conventional system with 30 pg/mL of IL6, which is the average serum concentration found in Stage III myeloma patients [19], every day for 3 days. As shown in Figure 4A, we found that the addition of IL6 led to a significant increase of the total viable CD38+ cell numbers in 3D culture on day 3. Specifically, in three of three MM samples, the treatment of IL6 led to a significant increase in viable CD38+ cell number from 41% to 68% in 3D culture (*p* = 0.021, paired Student’s *t*-test). On the other hand, no significant difference was observed in conventional culture with the presence of IL6 (*p* = 0.69, paired Student’s *t*-test). Correlating with these findings, we observed a significant increase of CD38 + pSTAT3+ cell number from 85% to 195% in the presence of IL6 in 3D culture on day 3 (*p* = 0.014, paired Student’s *t*-test, Figure 4B). No increase in CD38 + pSTAT3+ cell number was seen in the conventional culture after IL6 treatment (*p* = 0.737, paired Student’s *t*-test, Figure 4B). This result suggests that PM cells in 3D culture are more sensitive to IL6-induced STAT3 activation, which leads to further improved cell viability.

### 2.5. Stattic Inhibits Cell Growth of PM in 3D Culture but Not in Conventional Culture

**Figure 5:**
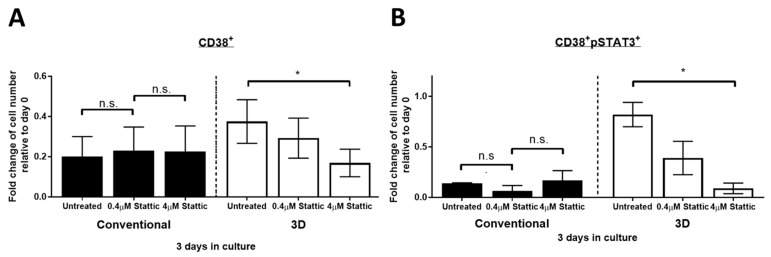
Stattic ablates PM cell viability in 3D culture but not in conventional culture. Fold change of viable (**A**) CD38+ and (**B**) CD38 + pSTAT3+ cell number from 3 MM patients in conventional or 3D culture on day 3 relative to day 0. Doses of 0.4 or 4 µM Stattic were added to cells on day 2 for 24 h. The mean value was indicated with error bars representing standard error. n.s. not significant, * *p* < 0.05, paired Student’s *t*-test.

Given the positive correlation between STAT3 activity and the survival of MM cells in 3D culture, we next asked if inhibition of STAT3 ablates MM cell viability. According to our previous study, compared to conventional culture, approximately 10 times higher drug dose is required to achieve equivalent drug-protein binding in 3D culture [14]. In keeping with this, we treated the bone marrow mononuclear cells from myeloma patients in 3D culture with two different doses of Stattic (0.4 and 4 µM) required to substantially bind to STAT3 in U266 cells cultured conventionally and in 3D, respectively [14]. As shown in Figure 5A, in three of three cases, the total number of viable CD38+ MM cells significantly decreased from 38% to 17% in 3D culture after treated with 4 μM Stattic for 24 h on day 3 (*p* = 0.038, paired Student’s *t*-test). On the other hand, the change of CD38+ cell number remained insignificant in conventional culture after the treatment of both 0.4 and 4 µM Stattic for 24 h on day 3 (*p* = 0.25 and *p* = 0.48, respectively, paired Student’s *t*-test). Consistent with these findings, the CD38 + pSTAT3+ cell number in 3D significantly decreased from 82% to 9% in the presence of 4 μM Stattic (*p* = 0.010, paired Student’s *t*-test). On the other hand, no significant change was seen in conventional culture in the presence of both 0.4 and 4 µM Stattic (*p* = 0.278 and *p* = 0.796, respectively, paired Student’s *t*-test) (Figure 5B). These results suggest that STAT3 activity contributes to superior MM cell viability in 3D culture but not in conventional culture. These findings also confirmed the importance of STAT3 in increasing the cell viability of PM cells grown in 3D.

## 3. Discussion

Primary patient cancer cells are generally regarded to be superior to cancer cell lines in the studies of cancer biology and therapeutics, since the former is more representative of the biology of the disease. Nonetheless, the vast majority of published myeloma studies employed cell lines. Our literature search has identified only a handful of recent myeloma studies employing PM cells. In a few studies in which both cell lines and PM cells were used, substantial discrepancies were identified. In one study, it was found that myeloma cell lines often overexpress Myc by gene amplification while such amplification is typically absent in PM cells [20]. The use of PM cells is typically limited by several factors. Procurement of patient samples can be a logistical challenge for many researchers. As pointed out earlier, PM cells are known to die shortly after being placed in conventional tissue culture. In one study, the viability of purified CD138+ PM cells decreased to 20% three days after being placed in conventional culture [11]. In accordance with this finding, we found that the average viability of PM cells on day 3 was 25% in this study. Taken together, these challenges have largely precluded the use PM cells for most biological assays.

Our review of the literature has revealed only a relatively small number of studies in which the primary goal is to increase the viability of PM cells ex-vivo. In one study using a single patient sample, the authors found that the addition of exogenous cytokines (including IL6, VEGF and IGF-1) and bone marrow stromal cells from healthy donors substantially improved the cell viability on day 7 from 500,000 to 200,000 cells [11]. Using a novel tissue culture system in which CD138+ PM cells harvested from four different patients were grown on a monolayer of human fetal bone marrow stromal cells, another research group successfully grew 30,000 cells to approximately 50,000 cells in 14 days [21]. A significant limitation of this study is related to the requirement of procuring marrow stromal cells, which were collected from the femurs of 8–12 weeks old human fetuses obtained after simultaneous abortions. In another study, researchers reported that the cell viability of CD138+ PM cells harvested from 38 patients cultured with 20% pooled plasma patient were significantly higher than those cultured with 10% fetal calf serum (60% of the original viable cells vs. 40%, day 3) [22]. However, the high diversity in components and low reproducibility by using pooled patient plasma are the major limitations of this study. Lastly, a microfluidic device was attempted to amplify PM cells but the results were not encouraging [23]. To our knowledge, none of these above-mentioned studies assessed the biology underlying the improvement of PM cell viability observed.

More recently, two research groups employing different 3D cell culture models to increase the viability of PM cells has been published [24,25]. In the first study, bone marrow mononuclear cells from 3 myeloma patients along with human endothelial cells were seeded into a 3D matrix composed of fibrinogen [24]. The cell proliferation was measured by the Ki-67 staining and analyzed by flow cytometry. It was found that the CD138+ PM cells in 3D cultured showed a 2.5-fold increase in cell proliferation in 7 days, whereas those in conventional culture did not change their proliferation rate. The second study employed matrigel-based 3D culture of CD138+ PM cells with osteogenic mesenchymal stromal cells, which was found to significantly increased the cell growth of PM cells, with a ~3-fold increase in the number of viable CD138+ cells in 3 days [25]. For both studies, we are concerned with the finding of a rapid rate of cell proliferation, which is contradictory to the biology of myelomas, which are typically non-proliferative tumors [26].

Compared to the above-mentioned 3D culture systems, Kirshner et al. introduced a more feasible model which recapitulates the stroma of bone marrow by ratiometrically mixing matrigel with collagen, laminin and fibronectin [13,27]. Kirshner et al. demonstrated that 3D cultured PM cells underwent cell proliferation, detectable by the disappearance of CFSE staining, for up to 25 days in four patients [13]. In contrast with other 3D models which require the co-culture with other cell types, Kirshner’s model allows preparation of 3D cultured MM cells for downstream molecular analyses (such as western blots and quantitative real-time PCR) without isolating PM cells [14]. While we adopted the 3D model that is published by Kirshner et al. [12], we employed a different approach to estimate the number of viable MM cells by combining both flow cytometric analysis of CD38+ percentage and trypan blue staining of viable bone marrow mononuclear cells. Moreover, we showed that this 3D model improved the survival of PM cells compared to conventional culture, a property that was not assessed by Kirshner et al. Additionally, we used a higher number of patients (n = 15) compared to Kirshner’s study (n = 4) with ranges of bone marrow cellularity, percentages of malignant plasma cells and cytogenetics.

In this study, we also have validated the relationship between STAT3 activity and PM cell viability in 3D culture. We found that induction or inhibition of STAT3 activity did not affect PM cell viability in conventional culture. This finding is different from what was found using the human MM cell line, U266, in conventional culture, where IL6 induces STAT3 activation and anti-apoptotic ability [28]. This discrepancy can be due to that not all MM patients have high expression of IL6 receptor which transmits the IL6 stimulating signal. Indeed, in one study it was reported that cell lines with low expression of IL6 receptor require both IL6 and insulin-like growth factor 1 together to stimulate MM cell growth [29]. Our previous study also demonstrated that STAT3 is activated in MM cell lines in 3D culture compared to conventional culture [14]. Moreover, blocking STAT3 induced apoptosis in 3D cultured MM cells but had no effect in conventionally cultured MM cells. Collectively, these findings suggest that STAT3-dependent survival of PM is dependent on the 3D environment.

The STAT3 activity in PM cells has been investigated with different parameters in three independent studies. Bharti et al. examined the activity of STAT3 using immunofluorescence staining of STAT3 and found that CD138+ cells from >50% of 22 MM patients showed strong nuclear STAT3 staining which implicated high STAT3 transcriptional activity [15]. In another study using flow cytometric analysis, it was shown that about five-fold more CD38+ cells were pSTAT3 (Y705)-positive in MM patients as compared to healthy donors [16]. The third study reported that around 50% of 48 cases showed pSTAT3 (Y705) positivity in CD138+ MM cells by immunohistochemical analysis [17]. The level of pSTAT3 (Y705) is positively correlated with poor progression-free survival and overall survival with hazard ratios of 3.7 and 3.5, respectively [30]. These findings suggest the essentiality of STAT3 activity in PM.

Some STAT3 inhibitors such as Icaritin and LLL12 have shown their ability to inhibit the cell growth of STAT3-active PM cells in conventional culture [31,32]. Our group has also developed several nanoparticular formulations of STAT3 inhibitors to improve biocompatibility, MM-targeting specificity and safety in-vivo [14,33,34]. However, these studies were performed in conventional culture and did not account for the loss of PM cell viability and STAT3 activity during STAT3 inhibitor treatment, which may underestimate of the potency of STAT3 inhibition. Our study highlighted the dependence on STAT3 activity for PM cells in the context of 3D environment, providing insight on targeting STAT3 and microenvironment as a promising strategy for treatment of MM.

In conclusion, our current study suggested the superiority of 3D culture in supporting the growth of PM cells. In addition, we demonstrated that 3D-cultured PM cells are dependent on their STAT3 activity for superior cell viability compared to conventional culture. Prolonged longevity of PM cells in 3D allowed long-termed gene manipulations and/or drug treatment for studying MM in a more realistic manner.

## 4. Materials and Methods

### 4.1. Patient Samples, 3D Culture and Stattic/IL6 Treatment

The characteristics of MM patients from which the bone marrow aspirates were extracted are outlined in Table 1. All procedures of patient sample handling were followed according to a protocol approved by Health Research Ethics Board of Alberta Cancer Committee (HREBA.CC-16-0346, approval date 5 December 2017). MM Bone marrow aspirates with signed patient consent form were collected from Cross Cancer Institute at the University of Alberta. Bone marrow mononuclear cells were extracted using a Ficoll-Paque gradient solution (GE Health Care, Chicago, IL, USA). The total viable cell number was estimated by trypan blue exclusion assay. The bone marrow mononuclear cells were subject to 3D culture as described previously [35]. In brief, bone marrow mononuclear cells were resuspended in reconstructed bone marrow matrix containing 4 parts of Matrigel^®^ (Corning, Corning, NY, USA), 2.5 parts of 1 mg/mL fibronectin and 1 part of 2 mg/mL collagen I at a concentration of 1 million cells per 100 µL. Then, 100 µL reconstructed bone matrix was loaded to each well on a 48-well plate pre-coated with reconstructed endosteum solution. After incubation at 37 °C for an hour, fresh bone marrow growth medium (BMGM, RPMI1460 medium supplied with 20% pooled human plasma from healthy donors) was added to the solidified reconstructed bone marrow matrix. Bone marrow mononuclear cells in 3D culture were harvested by dissolving in cell recovery solution (1× PBS with 5 mM EDTA, 1 mM Na3VO4 and 1.5 mM NaF solution). IL6 and Stattic lyophilized powder (Sigma, St. Louis, MO, USA) was dissolved in sterile water and DMSO, respectively, in a stock concentration of 10 μg/mL and 1 mg/mL, respectively. 3D cultured bone marrow mononuclear cells were treated with 30 pg/mL IL6 every day for 3 days or with 0.4 μM and 4 μM Stattic once on day 2 for 24 h.

### 4.2. Flow Cytometry Analysis

For CFSE staining, isolated bone marrow mononuclear cells were washed twice with DPBS (Gibco, Co Dublin, Ireland) and stained with 0.25 µM of CFSE (Invitrogen, Carlsbad, CA, USA) in DPBS for 20 min in dark at room temperature prior to seeding in 3D culture. The cells at different time points were recovered from the 3D culture using cell recovery solution and washed with PBS twice before flow cytometry analysis using FACSCanto II (BD Biosciences, San Jose, CA, USA). For CD38 staining, recovered 3D bone marrow mononuclear cells were washed in PBS twice and resuspended in 50 µL PBS containing 10 µL anti-CD38-PE (Santa Cruz) in dark at room temperature for 20 min. The cells were washed one time in PBS and subject to flow cytometry analysis using FACSCanto II. The same number of cells stained with isotype anti-mouse-PE were included as a reference control. For CD38-pSTAT3 double staining, recovered 3D bone marrow mononuclear cells were fixed in 2% paraformaldehyde at 37 °C for 10 min. The cells were washed in PBS with 1% BSA once and permeabilized with 100% methanol on ice for 30 min. The cells were then washed twice in PBS with 1% BSA and resuspended in 50 μL PBS with 10 μL anti-CD38-PE and 5 μL anti-pSTAT3-FITC (Santa Cruz, Santa Cruz, CA, USA) in dark at room temperature for 20 min. The cells were washed one time in PBS with 1% BSA and subject to flow cytometry analysis using FACSCanto II. The data was analyzed by FlowJo program version 10 (Becton, Dickinson & Company, Franklin Lakes, NJ, USA).

## Figures and Tables

**Table 1:** Characteristics of patients whose bone marrow cells were used in this study.

Patient #	Age	Gender	Diagnosis	Cytogenetic	% Bone Cellularity	% Plasma Cells	Anemia Present?	Light Chain Type	Notes
1	64	Male	Initial	CCND1	20–30	80	Yes	Kappa	-
2	78	Male	Initial	Gain CCND1 locus	50	12	No	Kappa	Plasmacytoma in ribs
3	52	Female	Initial	Not detected	60	80	Yes	Kappa	Plasmacytoma in femur
4	61	Female	Initial	Not detected	60–70	50–60	No	Kappa	-
5	45	Male	Initial	Not detected	80–90	90	Yes	Lambda	Plasmacytoma in ribs
6	63	Female	Initial	CCND1	80–90	70	Yes	Kappa	-
7	66	Female	Initial	Trisomy 17, CCND1	95	60	No	Lambda	Plasmacytoma in pelvis
8	55	Female	Relapse	CCND1	40	50	No	Lambda	-
9	73	Female	Initial	CCND1 and IgH, MAF	50	60	No	Lambda	-
10	54	Male	Initial	CCND1	50	30	No	Lambda	-
11	78	Male	Initial	Not detected	50	10	No	Lambda	-
12	66	Female	Initial	TP53 deletion	35	10–20	Yes	Lambda	Positive for amyloidosis, rib lesion
13	64	Male	Initial	Unknown	50	50	Yes	Kappa	Previous bone lesion
14	46	Female	Initial	Unknown	70–80	15	No	Lambda	-
15	77	Male	Initial	Unknown	60–70	70–80	No	Kappa	-

## Data Availability

Not applicable.
